# Slow Drinking of Beer Attenuates Subjective Sedative Feeling in Healthy Volunteers: A Randomized Crossover Pilot Study

**DOI:** 10.3390/nu14214502

**Published:** 2022-10-26

**Authors:** Shunji Oshima, Sachie Shiiya, Yasuhito Kato

**Affiliations:** Sustainable Technology Laboratories, Asahi Quality & Innovations, Ltd., 1-21, Midori 1-Chome, Moriya-shi 302-0106, Ibaraki, Japan

**Keywords:** alcohol, drinking speed, sedative feeling, beer, healthy volunteers

## Abstract

The change in physiological parameters and subjective feelings according to the speed of drinking alcohol has not been reported to date. The aim of this randomized crossover pilot study was to investigate the objective and subjective effects of different speeds of alcohol ingestion in healthy volunteers. Accordingly, 11 male and 7 female healthy Japanese adults were asked to consume 480 mL of beer at three different drinking speeds (80, 40, and 20 mL/5 min). According to the objective measurement, the transient increase in blood alcohol and serum uric acid concentrations was most inhibited at a drinking speed of 20 mL/5 min. Acetate, lactate, pyruvate, and lactate/pyruvate ratios did not differ between the three drinking speeds. Stimulant feelings measured by the subjective scores of the Brief Biphasic Alcohol Effects Scale did not differ between the three speeds. However, the sedative feeling score obtained at a drinking speed of 20 mL/5 min (the slowest speed of alcohol consumption) was significantly weakened in comparison with those obtained at drinking speeds of 40 and 80 mL/5 min. Therefore, a slower consumption of alcohol mitigated the subjective sedative feeling. The effects of slower alcohol consumption may be caused by the slower slope of the increasing trend of blood alcohol concentration.

## 1. Introduction

Alcohol consumption causes various physiological and pharmacological effects in the human body that can lead to many health problems [1,2,3]. Additionally, daily alcohol consumption affects subjective response [4,5,6]. A dose–effect relationship exists between these effects and blood alcohol concentration (BAC), as reflected by behavioral changes, reduced cognition, and impaired psychomotor performance [7]. Therefore, BAC regulation is important to attenuate the harmful effects of alcohol drinking on the body. BAC is influenced by the amount of alcohol consumed. In addition, some exogenous drinking conditions, such as the type of beverage (concentration of alcohol content) [8] and drinking with or without food [9,10,11], influence BAC, despite consuming the same amount of alcohol. The mechanisms involved are mainly related to the absorption process of alcohol. Drinking a more concentrated beverage, such as vodka/tonic, produces a higher peak BAC than drinking a less concentrated beverage, such as wine or beer, regardless of the total amount consumed [8]. Therefore, less concentrated alcohol is absorbed more slowly than more concentrated alcohol. Alcohol is also absorbed more slowly in individuals who have eaten than in those who have fasted, owing to the delay of the gastric emptying rate of alcohol [12]. Thus, BAC is lower in the fed state than in the fasting state after alcohol consumption [9,10,11]. Moreover, alcohol significantly impairs dual task performance, although the impairment is significantly reduced in individuals who had eaten before alcohol consumption [13].

Considering the acute harmful effects of alcohol, drinking slowly is recommended. Under conditions of slow drinking, alcohol is absorbed more slowly, possibly reducing the BAC [14]. However, the effects of slow alcohol consumption on BAC or physiological parameters remain unreported. Therefore, the aim of this this randomized, nonblind, crossover pilot study was to evaluate the effects of the speed of alcohol ingestion on physiological responses in healthy volunteers. Specifically, we sought to examine whether differences in the speed of consuming certain amounts of beer affect BAC, the biochemical indices affected by alcohol, and subjective stimulant or sedative feelings. Therefore, the results obtained in this study provide insights regarding slow drinking.

## 2. Materials and Methods

### 2.1. Volunteers

This clinical trial conformed to the principles of the Declaration of Helsinki, and the protocol was approved by the Suda Clinic Institutional Review Board (approval number: 2021-032). Volunteers were recruited from employees working at Asahi Group Research and Development Center (Ibaraki, Japan). Written informed consent was obtained from all participants. The inclusion criteria were as follows: healthy Japanese men and women aged 20–64 years and provision of written informed consent prior to participation. Conversely, volunteers who could not drink 480 mL of beer were excluded. Ultimately, 18 participants (men: *n* = 11, women: *n* = 7) were enrolled.

### 2.2. Study Design

**Figure 1:**
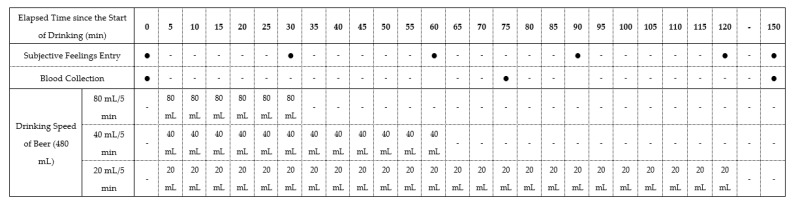
Time schedule of the experiments. Participants drank 480 mL of beer at three different speeds (80, 40, and 20 mL/5 min).

In this study, we used a nonblind, single-center, randomized crossover design. The study registered with the University Hospital Medical Information Network (UMIN-CTR ID: UMIN000045190). The 18 participants were randomly allocated to groups 1, 2, and 3, with 6 participants per group. All experiments for three patterns of drinking speed (drinking in 30, 60, and 120 min) were conducted in all groups in a random order in the sequence summarized in Table 1, with more than 1 week of washout period in between. Throughout the three experiments, all participants were instructed to maintain daily eating and drinking habits. Figure 1 presents the time schedule of the experiments.

A commercially available beer (5.0% alcohol content indicated on the product label), containing carbohydrates was used as the test beverage to avoid the induction of hypoglycemia [15] due to alcohol ingestion in a fasting state. Briefly, peripheral blood samples were collected from the cubital vein, and subjective drunkenness feeling was measured using the Brief Biphasic Alcohol Effects Scale (B-BAES) under a fasting state. Thereafter, the participants drank a total of 480 mL of beer at three different speeds (80, 40, and 20 mL/5 min), finishing drinking in 30, 60, and 120 min, respectively. Eating foods and drinking beverages other than the test beer were not allowed. However, participants were permitted to freely consume water after finishing drinking the beer. Blood samples were collected at 75 and 150 min, and B-BAES was recorded every 30 min.

### 2.3. Blood Analyses and Subjective Sensation Assessments

Following blood collection, we mixed 0.5 mL of blood samples with 0.5 N of perchloric acid (2.5 mL) and prepared supernatants by centrifugation. Subsequently, BAC was measured by headspace gas chromatography according to the procedures described by Okada and Mizoi [16]. We also measured blood acetate, lactate, and pyruvate concentrations by high-performance liquid chromatography (Shimadzu organic acid determination system; Shimadzu, Japan) with an ion-exclusion column and a conductivity detector [17]. Furthermore, serum levels of alanine aminotransferase, aspartic aminotransferase, gamma-glutamyl transferase, lactate dehydrogenase, creatine kinase, alkaline phosphatase, total bilirubin, triglyceride, uric acid, total protein, albumin, and glucose were measured by a Fuji Drychem 7000 system (Fujifilm Co., Tokyo, Japan).

Alcohol produces both stimulant and sedative sensations [18,19,20]. Martin et al. developed the BAES, which is a self-reporting measure of the impact of alcohol [12]. BAES is a reliable and valid 14-item measure of the acute stimulant and sedative effects of alcohol; however, its length may preclude its use in research paradigms with time constraints on assessment. Thus, in the present study, we used the six-item B-BAES [21,22]. The brief stimulation subscale of the B-BAES consists of energized, excited, and up, whereas the brief sedation subscale consists of sedated, slow thoughts, and sluggish. Each participant answered each item as a subjective sensation on a 10 cm visual analog scale. The distance (cm) from the left edge of the line to the mark placed by the participant was measured at each time point. This measurement was then used in subsequent analyses as the sensation score from 0 (not at all) to 10 (extremely) that best described their present feelings. To determine the stimulant or sedative score, we added the score of each of the three items of the stimulation or sedation subscale. The scores are expressed as the changing scores obtained from before drinking beer (0 min). In addition, we used the trapezoidal rule to calculate the area under the curves (AUC_0–150_) from 0 to 150 min of the score–time curves of subjective stimulant and sedative feelings.

### 2.4. Statistical Analysis

All statistical data were analyzed using BellCurve for Excel 3.20 (Social Survey Research Information, Tokyo, Japan). Temporal changes from 0 min to 150 min of each blood or serum concentration were analyzed for each variable using repeated-measures ANOVA. Differences among the three drinking speeds of the maximal changes in each blood or serum parameter and B-BAES scores, as well as the AUC_0–150_ of the scores, were analyzed using one-way ANOVA followed by Bonferroni post hoc test as paired analyses of parametric data; different letters in the figures indicate statistically significant differences. These data are presented as means ± standard deviation. Pearson’s simple linear regression analysis was used to evaluate the correlations of maximal blood alcohol concentration (BAC-max) with each maximal score of subjective feeling. A value of *p* < 0.05 was considered significant.

## 3. Results

All participants underwent three experiments for the three drinking speeds. Final analyses were conducted using data from 18 healthy participants aged 36.3 ± 9.9 years. Table 2 lists the characteristics of the participants, and Table 3 summarizes the temporal changes, the maximum amount of change from 0 min, and the AUC_0–150_ of each blood or serum parameter. All drinking speeds indicated a significant temporal change in all parameters. The maximal change in BAC at the drinking speed of 20 mL/5 min was significantly lower than that at 80 and 40 mL/5 min 75 min after the start of drinking (*p* < 0.001 and *p* < 0.001, respectively), although the BAC values 150 min after the start of drinking were similar among the three drinking speeds. The maximal changes in acetate, lactate, pyruvate, and lactate/pyruvate ratios were not significant among the three drinking speeds. The maximal changes in uric acid concentrations at a drinking speed of 20 mL/5 min were significantly lower than at drinking speeds of 40 and 80 mL/5 min (*p* < 0.001 and *p* = 0.015, respectively).

**Figure 2:**
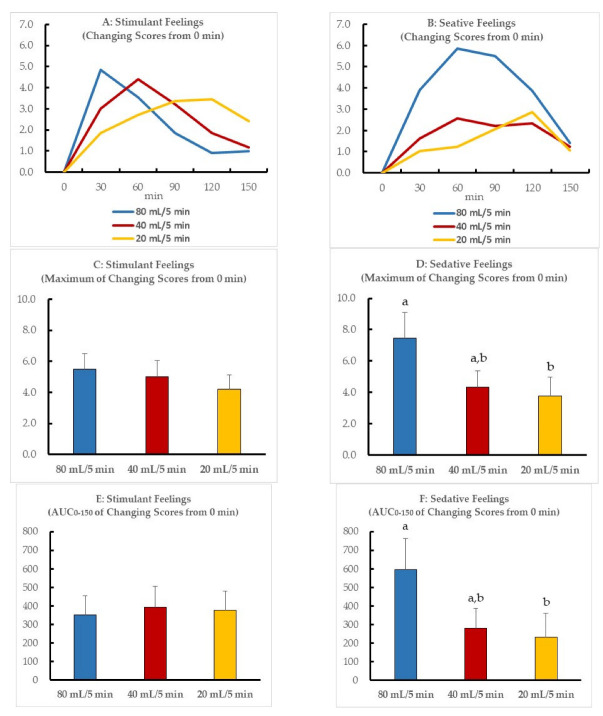
(**A**) Temporal changes in the mean sum of the three scores of the stimulation subscale (Energized, Excited, and Up) are indicated as mean values. (**B**) Temporal changes in the mean sum of the three scores of the sedation subscale (Sedated, Slow thoughts, and Sluggish) are indicated as mean values. The scores of the subjective stimulant or sedative feelings reported by the volunteers after beer ingestion are expressed as means and standard deviation ((**C**) maximal scores of stimulant feelings, (**D**) maximal scores of sedative feelings, (**E**) AUC_0–150_ of temporal scores of stimulant feelings, (**F**) AUC_0–150_ of temporal scores of sedative feelings). Scores with different letters (a,b) are significantly different among the three drinking speeds at *p* < 0.05 according to the Bonferroni test.

Figure 2 illustrates the temporal changes in scores, maximal scores, and the AUC_0–150_ of the stimulant and sedative feelings. The time that reached the maximal score differed between the three drinking speeds. The stimulant feelings increased until finishing drinking alcohol and decreased thereafter (Figure 2A); however, the maximal and AUC scores were did not significantly differ between the three drinking speeds (Figure 2C,E). Conversely, the scores of sedative feelings showed differences between the three speeds (Figure 2B). The maximal and AUC scores at a drinking speed of 20 mL/5 min were significantly lower than those at a drinking speed of 80 mL/5 min (Figure 2D,F), but those at a drinking speed of 40 mL/5 min did not significantly differ from those at 80 and 20 mL/5 min.

Table 4 illustrates the relationship of maximal blood alcohol concentration (BAC-max) with each maximal score of subjective feeling. There was no relationship between BAC-max and each score of stimulant feelings (energized, excited, up, or stimulant). On the other hand, the scores of sedated, slow thoughts, and sedative had a significant positive relationship with BAC-max (*p* = 0.003, 0.046, and 0.042, respectively).

## 4. Discussion

We examined whether differences in the speed of consuming 480 mL of beer affected BAC, biochemical indices, and subjective feelings in a crossover pilot trial in 18 healthy subjects. Considering that the objective and subjective effects caused by alcohol consumption vary widely among individuals, a crossover study was employed to ensure that participants’ backgrounds did not differ when comparing the effects of different drinking speeds. Results showed that BAC transition in the consumption of 480 mL of beer for 30 min was almost the same as that for 60 min. At a drinking speed of 20 mL/5 min, although the participants had finished 280 mL after 75 min, the BAC (0.13 mg/mL) was only less than half of that (0.34 mg/mL) at the other drinking speeds. Smith et al. observed increasing maximal BAC and BAC-AUC with increasingly rapid portal-vein infusions of identical alcohol doses in rats [23]. The BAC in portal blood to the liver could be an important determinant of maximum BAC and BAC-AUC, owing to the first-pass metabolism of alcohol in the liver. If the absorption dose of alcohol is sufficiently low to maintain the portal BAC below the Vmax of hepatic alcohol dehydrogenase, some ethanol will escape into the peripheral circulation, resulting in very low BAC and AUC. The drinking speed of 20 mL/5 min also lowered the maximal BAC by causing lower portal BAC resulting from the lower absorption amount per time of alcohol consumption.

Acetate is a metabolite of ethanol, and the lactate/pyruvate ratio, which reflects NADH/NAD+ ratio as a redox state, transiently elevates after alcohol ingestion [24]. Despite differences in BAC values, the blood acetate, lactate and pyruvate concentrations, and lactate/pyruvate ratio did not significantly differ between the three drinking speeds. None of the changes in organic acids that occurred after alcohol consumption were affected by differences in the drinking speed. A small amount of alcohol could sufficiently affect these parameters. After alcohol consumption, the urinary output of uric acid is reduced, thereby transiently increasing serum uric acid concentration [25]. Of the three drinking speeds, 20 mL/5 min resulted in the smallest increase in uric acid concentrations; therefore, drinking alcohol more slowly may have a limited effect on serum uric acid levels with lower BAC. Alcohol is recognized a risk factor for increased uric acid and gout flare [26]. Drinking slowly may be recommended for the prevention of gout.

Alcohol consumption reportedly increases the subjective stimulation scores during the initial increase in BAC and increases subjective sedation scores during the declining phase of BAC values [19,27]. The present study revealed that at a drinking speed of 80 mL/5 min, stimulant feelings were maximal at the end of drinking, and the sedative feelings reached maximum values at 60 min. However, at a slower speed of drinking, both scores tended to decrease as the participants finished drinking, possibly as a result of the suppression of the rapid increase in BAC. Surprisingly, the difference in the maximum stimulant effects between the three drinking speeds was not significant, despite a lower maximal BAC at a drinking speed of 20 mL/min. In contrast, the sedative feeling was significantly lower at a drinking speed of 20 mL/5 min than that at 80 mL/5 min. We confirmed the order effect of the sedative feeling score (maximal score and AUC), which indicated a significant difference among three drinking speeds in the crossover design. As a result, the order effect was not shown in the sedative data. When the correlation between BAC and the scores of stimulant feelings was examined, the stimulant feeling did not show a correlation with the BAC, but the scores of sedative feelings showed a significant positive correlation, proving that the intensity of stimulant feelings did not depend on the degree of BAC elevation. A previous report indicated that stimulant feeling was similar between 0.2 g/kg and 0.4 g/kg of alcohol ingestion, but sedative feeling was significantly stronger with 0.4 g/kg of alcohol than 0.2 g/kg of alcohol when healthy adults ingested moderate amounts of alcohol [28]. The results of the present study are in agreement with that of the abovementioned study. Thus, by drinking slowly, a similar stimulant feeling was obtained as in the case of drinking quickly, but the sedative feeling was less likely to occur. The present study is the first to demonstrate that drinking slowly produces subjective stimulant feelings while suppressing subjective sedative feelings. However, further research is needed because the effect on the subjective drinking sensation may vary depending on individual drinking conditions. Drinking slowly may result in lower total BAC, and the recommendation to do so was considered to be correct in terms of physical and psychological effects. Stafford et al. found that alcoholic beverages were consumed at a slower rate in two health warning conditions compared with the control condition [29]. However, realistically, 480 mL of beer would be unlikely to be consumed over an hour as in this study. Therefore, drinking conditions should be devised. For example, selecting alcoholic beverages with a lower alcohol content instead of changing the drinking speed of alcohol would be practically the same as slowly consuming alcohol. To the best of our knowledge, our study findings are the first to suggest health effects resulting from the difference in speed of consuming the same amount of alcohol.

This study is subject to some limitations. First, a power analysis for calculating the required number of study participants was not performed during the planning phase of the study. The modest sample size (*n* = 18) might have reduced the statistical power and increased the risk of type II error, particularly regarding the statistical differences in multiple comparisons between the sedative feelings at drinking speeds of 40 and 80 mL/5 min. Evidence of effects caused by the difference in speed of consuming alcohol could be acquired by conducting clinical trials with an appropriate sample size. Furthermore, differences in the amount of alcohol consumed could also affect the results, although a moderate amount of beer (480 mL) was consumed in this study. Additional studies are required to clarify the health effects resulting from the difference in speed of alcohol consumption.

## 5. Conclusions

We confirmed that drinking slowly moderates the increase in BAC. Drinking slowly did not change the stimulant feeling but weakened the sedative feeling. This practice also suppressed the transient increase in serum uric acid concentration. Results suggest that these changes were caused by the slower slope of the increasing trend of BAC resulting from slow alcohol drinking. Drinking slowly could reduce the objective and subjective acute effects of alcohol; hence, slow drinking is recommended. Further studies are required in the future.

## Figures and Tables

**Table 1:** Study schedule showing the nonblind, single-center, randomized crossover pilot study design.

	1st Experiment	Washout (>1 Week)	2nd Experiment	Washout (>1 Week)	3rd Experiment
Group 1 (*n* = 6, ID: 1–6)	20 mL/5 min	→	80 mL/5 min	→	40 mL/5 min
Group 2 (*n* = 6, ID: 7–12)	40 mL/5 min	→	20 mL/5 min	→	80 mL/5 min
Group 3 (*n* = 6, ID: 13–18)	80 mL/5 min	→	40 mL/5 min	→	20 mL/5 min

Arrows indicate washout periods (>1 week).

**Table 2:** Characteristics of the healthy participants (*n* = 18).

Parameter	Mean (SD)
Age (years)	36.3 (9.9)
male, *n* = 11	40.1 (9.9)
female, *n* = 7	30.4 (6.3)
Height (cm)	166.7 (7.9)
male, *n* = 11	171.7 (4.0)
female, *n* = 7	158.9 (6.0)
Body weight (kg)	61.0 (8.6)
male, *n* = 11	66.1 (6.1)
female, *n* = 7	53.0 (5.1)
Body mass index (kg/m^2^)	21.9 (2.0)
male, *n* = 11	22.4 (2.0)
female, *n* = 7	21.0 (1.5)
ALT (U/L)	18.6 (7.6)
AST (U/L)	23.0 (4.0)
GGT (U/L)	28.2 (18.2)
LDH (U/L)	174.6 (25.6)
CK (U/L)	144.1 (63.2)
ALP (U/L)	193.4 (44.6)
Total bilirubin (mg/dL)	0.7 (0.3)
Triglyceride (mg/dL)	80.6 (49.9)
Uric acid (mg/dL)	6.0 (1.2)
Total protein (g/dL)	7.3 (0.5)
Albumin (g/dL)	4.9 (0.4)
Glucose (mg/dL)	80.3 (5.7)

Data are presented as mean (standard deviation). Abbreviations: ALT, alanine aminotransferase; AST, aspartic aminotransferase; GGT, gamma-glutamyl transferase; LDH, lactate dehydrogenase; CK, creatine kinase; ALP, alkaline phosphatase.

**Table 3:** Temporal changes and maximum amount of change from 0 min of each blood or serum parameter concentration in the crossover trial.

Parameter	Drinking Speed (mL/5 min)	0 min	After 75 min	After 150 min	*p ^c^*	Maximum
Alcohol (mg/mL)	80	0.0	0.34 (0.07) ^a^	0.17 (0.07)	<0.001	0.34 (0.07) ^a^
	40	0.0	0.34 (0.08) ^a^	0.17 (0.06)	<0.001	0.34 (0.08) ^a^
	20	0.0	0.13 (0.04) ^b^	0.17 (0.06)	<0.001	0.18 (0.05) ^b^
Acetate (μg/mL)	80	0.0	18.1 (6.4)	16.0 (7.8)	<0.001	19.6 (7.2)
	40	0.0	20.4 (9.3)	18.1 (6.4)	<0.001	22.1 (8.6)
	20	0.0	20.3 (9.0)	15.5 (5.8)	<0.001	21.1 (8.5)
Lactate (mg/dL)	80	15.8 (5.0)	19.7 (4.3)	17.5 (4.6)	0.007	3.3 (6.7)
	40	13.5 (6.8)	19.4 (4.1)	19.5 (7.1)	<0.001	7.5 (8.1)
	20	13.9 (5.5)	18.5 (3.7)	18.3 (6.5)	0.027	5.6 (7.3)
Pyruvate (mg/dL)	80	1.00 (0.25)	0.51 (0.12)	0.47 (0.11)	<0.001	−0.55 (0.25)
	40	0.90 (0.34)	0.48 (0.12)	0.53 (0.25)	<0.001	−0.43 (0.44)
	20	0.84 (0.25)	0.48 (0.14)	0.48 (0.17)	<0.001	−0.41 (0.26)
Lactate/pyruvate ratio	80	15.6 (3.9)	38.5 (9.2)	37.4 (9.4)	<0.001	27.1 (9.9)
	40	14.4 (3.0)	40.7 (8.3)	39.0 (10.6)	<0.001	30.9 (10.2)
	20	16.1 (4.0)	39.0 (8.9)	38.8 (9.7)	<0.001	28.0 (10.4)
Uric acid (mg/dL)	80	5.8 (1.3)	6.1 (1.3)	6.0 (1.3)	<0.001	0.3 (0.1) ^a^
	40	5.7 (1.4)	6.1 (1.4)	6.0 (1.5)	<0.001	0.4 (0.2) ^a^
	20	5.8 (1.2)	6.0 (1.2)	6.0 (1.2)	<0.001	0.2 (0.2) ^b^

Data are presented as mean (standard deviation). The means of the blood alcohol concentrations (BAC) and the maximum amount of change in BAC or serum uric acid concentration with different superscript letters (^a,b^) were significantly different between the three drinking speeds (*p* < 0.05) according to the Bonferroni test. *^c^* Temporal changes from 0 min to 150 min of each blood or serum concentration were analyzed for each variable using repeated-measures ANOVA.

**Table 4:** Relationship of BAC-max with each maximal score of subjective feeling in the crossover trial.

	Stimulant Feelings				Sedative Feelings			
	Energized	Excited	Up	Stimulant	Sedated	Slow Thoughts	Sluggish	Sedative
BAC-max	0.010	−0.096	−0.023	−0.037	0.394 **	0.273 *	0.014	0.278 *

Data are presented as correlation coefficients to explore the relationship of BAC-max with each maximal score of subjective feeling (*n* = 54, * *p* < 0.05, ** *p* < 0.01). Stimulant score is a sum of three scores (energized, excited, and up). Sedative score is a sum of three scores (sedated, slow thoughts, and sluggish).

## Data Availability

The datasets used in this study are available from the corresponding author upon reasonable request.
